# Sparsity-based Ankylography for Recovering 3D molecular structures from single-shot 2D scattered light intensity

**DOI:** 10.1038/ncomms8950

**Published:** 2015-08-20

**Authors:** Maor Mutzafi, Yoav Shechtman, Yonina C. Eldar, Oren Cohen, Mordechai Segev

**Affiliations:** 1 Physics Department and Solid State Institute, Technion, Haifa 32000, Israel; 2 Department of Chemistry, Stanford University, 375 North-South Mall, Stanford, California 94305, USA; 3 Electrical Engineering Department, Technion, Haifa 32000, Israel

## Abstract

Deciphering the three-dimensional (3D) structure of complex molecules is of major importance, typically accomplished with X-ray crystallography. Unfortunately, many important molecules cannot be crystallized, hence their 3D structure is unknown. Ankylography presents an alternative, relying on scattering an ultrashort X-ray pulse off a single molecule before it disintegrates, measuring the far-field intensity on a two-dimensional surface, followed by computation. However, significant information is absent due to lower dimensionality of the measurements and the inability to measure the phase. Recent Ankylography experiments attracted much interest, but it was counter-argued that Ankylography is valid only for objects containing a small number of volume pixels. Here, we propose a sparsity-based approach to reconstruct the 3D structure of molecules. Sparsity is natural for Ankylography, because molecules can be represented compactly in stoichiometric basis. Utilizing sparsity, we surpass current limits on recoverable information by orders of magnitude, paving the way for deciphering the 3D structure of macromolecules.

## Abstract (web summary)

X-ray laser holds promise for deciphering three-dimensional structures of organic molecules, which constitutes a major goal in structural biology. Mutzafi *et al.* propose an algorithm to overcome the issue of laser-induced sample damage based on prior knowledge of the atoms that comprise the molecules.

## Graphical abstract

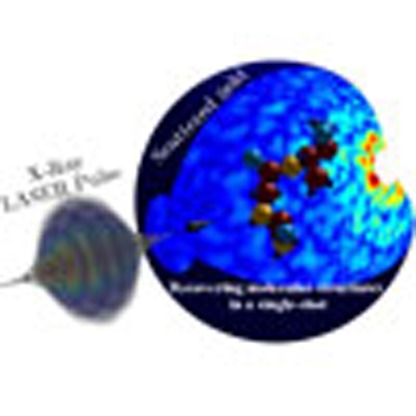

Recovering the three-dimensional (3D) structure of biological molecules is of paramount importance. For example, protein characterization plays a key role in the field of structural proteomics^1^,^2^,^3^. Knowing the protein structure may provide further understating of the function and mechanism even for proteins whose biochemical function is known^4^. The main methodology used today to recover 3D structure of molecules is X-ray crystallography, which requires crystallization of the probed molecules. This method relies on X-ray diffraction from a periodic structure, which averages over many molecules making up the ‘molecular crystal'. However, the molecules in such a structure are not situated in the same exact position and alignment in all unit cells, hence this method fundamentally cannot provide sufficient resolution in the recovered 3D structure of the molecule. Moreover, there is an additional even greater problem: while small molecules (having few degrees of conformational freedom) may be crystallized by various methods, such as chemical vapour deposition^5^ and re-crystallization^6^, for macromolecules, especially membrane proteins, crystallization is much more problematic^7^. In fact, thus far, crystallization attempts have been unsuccessful for most of the membrane proteins; as such, the 3D structure of many bio-molecules is still unknown^7^. Clearly, developing a method that could decipher the 3D structure of a single protein molecule is nothing less than a dream. In fact, no current method can do that even in theory.

In the past few years, it has been proposed to study such molecules using imaging with X-ray laser pulses^6^,^8^,^9^,^10^, whose wavelength has the desired resolution. However, since X-ray light ionizes all biological molecules and changes their molecular structure, X-ray experiments on organic molecules cannot be carried out with continuous wave (CW) radiation. Rather, this has to be performed with ultrashort laser pulses. Moreover, biological molecules disintegrate after the first pulse, and therefore the information (scattered light) necessary for recovering the structure must be collected either in a single shot (the basis of Ankylography), or in multiple shots—each probing a new molecule of the same kind, followed by a calibration procedure (registration) since the molecule in each shot is inevitably rotated in 3D space. Such ideas have indeed been suggested^11^,^12^,^13^. Experimentally, there were successful attempts using single-shot X-ray pulses scattered off aerosol particles, demonstrating the ability to determine the orientation of two large polystyrene spheres^14^ and finding the two-dimensional (2D) projection of several particles^15^. Going back to a single biological molecule, when a single ultrashort X-ray pulse is launched at such a molecule, and when the pulse is short enough—the flux of photons scattered off the molecule before it disintegrates carries the information about the structure^16^,^17^. The 3D structure of the molecule can then be recovered algorithmically from this single measurement, in a process called Ankylography^10^. This approach for deciphering the molecular structure relies on the ability to use ultrashort (femtosecond) laser pulses in the X-ray regime. Indeed, recent developments have enabled the construction of a new X-ray free electron laser (XFEL) facility, which emits a beam with high coherence and facilitates access to atomic scale imaging^18^,^19^. In fact, the wavelength of X-ray laser flashes are so short that even atomic details may one day become discernible (*λ*∼0.5 Å–6 nm). Another source of ultrashort X-ray pulses is based on the high-harmonic generation process, which already enables coherent experiments in the X-ray regime^20^.

Desirably, the coherent scattering measurements should be taken at the surface corresponding to the Ewald sphere^21^ (a sphere in the Fourier domain; see explanation in the Supplementary Information). But even in this case, such a single-shot 2D measurement is still missing a very large part of the information necessary to recover the 3D structure. Namely, the phase information is missing, and only 2D data is obtained. Therefore, Ankylography describes an algorithmic procedure whose goal is to recover 3D information from a single-shot magnitude-only measurement taken on a 2D surface corresponding to the Ewald sphere of the sought information. The algorithmic methodology of Ankylography relies on phase-retrieval algorithms, known for several decades^22^,^23^, which have recently found their way into applications with coherent X-ray radiation^24^,^25^. Still, achieving phase-retrieval for 3D structures from 2D measurements, as Ankylography is attempting to do, is a formidable challenge.

In spite of these problems in trying to recover the 3D structure from highly incomplete measurements, a visionary proof-of-concept Ankylography experiment has recently been demonstrated, attracting much interest^10^. However, the excitement has not been unanimous among researchers. For example, in a recent exchange in *Nature* magazine researchers compared the idea, to pulling a 3D rabbit out of a 2D hat^26^. The original Ankylographic method was believed to work for only objects containing <15^3^ voxels (volumetric picture element)^26^,^27^, but actually the original paper has demonstrated the recovery of larger objects, with the current state of the art being 32 × 32 × 20 voxels. While researchers have not yet reached a consensus on exact limits of Ankylography^10^, serious doubts were cast on its feasibility^28^,^29^,^30^, uniqueness and stability^31^,^32^. Moreover, it was claimed that, Ankylography will not work in the absence of additional constraints^28^. Notwithstanding these important arguments, recent experiments have demonstrated good progress in Ankylography, but all under stringent assumptions on the symmetry of the recovered structures^33^ or multiple measurements^34^.

Here, we propose and numerically demonstrate a new algorithmic paradigm for reconstructing 3D objects from their scattered 2D intensity. Our approach is based on sparsity: prior knowledge that the information is sparse in a known basis. In our context, sparsity is manifested in the fact that the molecule effectively occupies small number of degrees of freedom (d.f.) (because molecules are made of atoms), and that the chemical composition (stoichiometry) of the molecule is known. As such, the prior knowledge of sparsity can be utilized to recover the ‘signal' from highly incomplete measurements. Using recently developed algorithmic tools for sparsity-based phase retrieval^35^, we demonstrate numerically the ability to determine the atomic structures of various complex organic molecules, such as peptides. This illustrates that sparsity and optimization techniques enable surpassing current limits on the recoverable information in Ankylography, by orders of magnitude. We test the performance of our methodology with respect to sparsity (number of atoms) and noise, and conclude that sparsity can pave the way to algorithmic reconstruction of the 3D structure of molecules from a single measurement of the photon flux in the optical far field.

## Results

### The sparsity-based concept

Before going into the mathematical details of sparsity-based Ankylography, let us explain the logic of our approach and its background. In Ankylography, a major part of the information is lost due to physical limitations, which leads to dimension deficiency and to lack of phase information in the measured data. In the most general case, it is possible to recover 3D information by taking multiple projection measurements and appropriate signal processing. Common methods to do that include computed tomography^36^ (CT), equally sloped tomography^37^ and more. However, here, traditional methods to recover 3D information from 2D measurements cannot be employed, because they require multiple measurements from different projections, while in the current physical problem, multiple projections are extremely hard (if not impossible) to realize in experiments. This is the motivation for Ankylography: attempting to rely strictly on data acquired in a single-shot experiment, in spite of the fact that a large part of the information is missing in the measurements. This is where sparsity comes into play. As we show below, the underlying problem typically features only a small number of d.f.. As such, our use of sparsity is natural, relying on the logic of our earlier work on sparsity-based subwavelength imaging^38^,^39^ and super-resolution^40^,^41^,^42^, and on sparsity-based phase retrieval^35^,^39^,^41^,^42^. The theoretical framework underlying the recovery procedure is borrowed from the emerging field of compressed sensing^43^,^44^,^45^. The main theme of compressed sensing is to reduce the number of acquired measurements of a signal, while still being able to accurately recover it by relying on the fact that the signal is described by a small number of d.f. Here, our goal is to recover the complete information from an inherently incomplete set of (quadratic) measurements. To this end, we adapt the recently proposed sparsity-based phase-retrieval technique (called GESPAR^35^) to our setting.

### Physical setting and sparse representation of the physical signal

**Figure 1:**
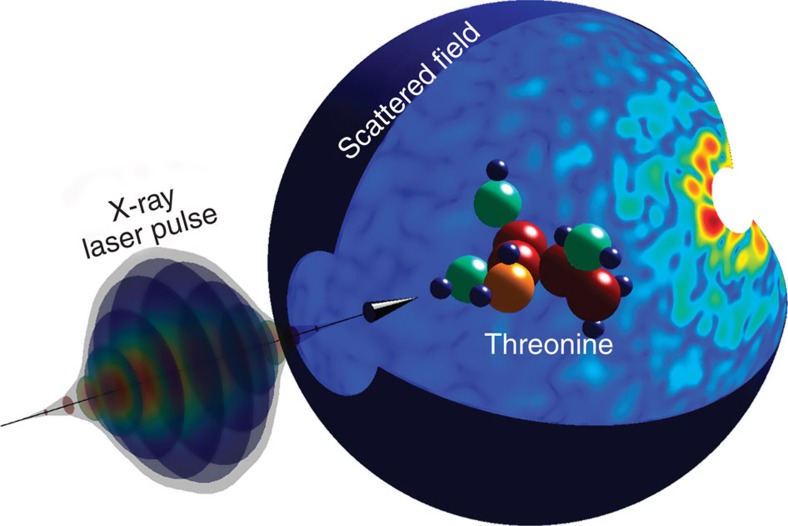
The physical setting for Ankylography. An ultrashort X-ray laser pulse is incident on a molecule, which scatters the photons before it disintegrates. The intensity of the scattered light is measured on a sphere in the optical far field, and from it the 3D structure of the molecule is computed.

The general physical setting for Ankylography is illustrated in Fig. 1. A coherent ultrashort laser pulse of central wavelength *λ* and relatively narrow bandwidth *δλ* (such that 
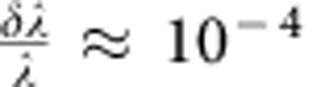
) is incident upon a molecule with a 3D structure we wish to recover. The light is scattered from the molecule within the ultrashort duration of the pulse (a few femtoseconds), but immediately thereafter the molecule disintegrates, such that the only measurements available are those taken from the scattered light in this single-shot experiment. The detectors can be positioned on the Ewald sphere, or more practically, use a planar camera and correct for the curvature. The 3D effective potential (core electron charge density) of the molecule, which is the source for X-ray scattering, can be described as a sum over known basis functions. For simplicity, we describe each atom as a sphere with its covalent radius^46^, although a more mathematically accurate description would be provided by a set of known, spherically symmetric functions^47^. It is important to note that, in spite of the fact that we described the molecule with simple basis functions, our methodology is general, and is not limited to a particular basis (see example in Fig. 5). In this scheme, the molecule resembles a set of hovering spheres. The 3D scattering potential of the molecule is given by the scalar function


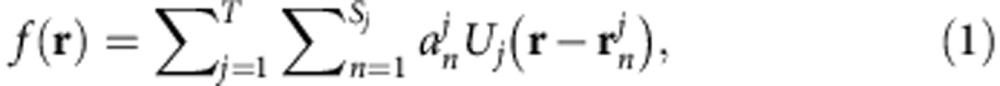


where the first summation is over the *T* kinds of elements comprising the molecule, and *U*_*j*_(**r**) is the potential related to the *j*-th element. The second summation is over *S*_*j*_, the number of atoms of the *j*-th element, with 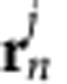 and 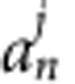 being the 3D position and amplitude of the *n*-th atomic wavefunction of the *j*-th element. Physically, 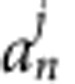 manifests the charge density in the core electrons of that atom, which is what scatters X-ray radiation^46^. For example, *U*_1_ reflects the potential of the 1st element in the molecule (say, carbon), while 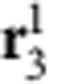 and 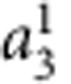 are the 3D position and the amplitude of the third carbon atom. Importantly, the chemical composition of the molecule is known (number of atoms of each element) from stoichiometry, as well as the covalent radius associated with each element. Hence, the only unknowns are the relative positions of the atoms, as described by the centre positions of the spheres, 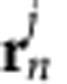, and the amplitudes 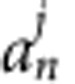. Altogether, the number of unknowns in the problem is relatively small, which is why sparsity-based methods can be very effective.

The scattered light intensity, which corresponds to the measured data, resides on a spherical surface of a large radius centred on the molecule, in the far field of the 3D image. Theoretically, to first order in perturbation theory^21^ the scattered field intensity is proportional to the 3D Fourier transform absolute value squared of the scattering potential, measured on the surface of a sphere called ‘the Ewald sphere'^21^:


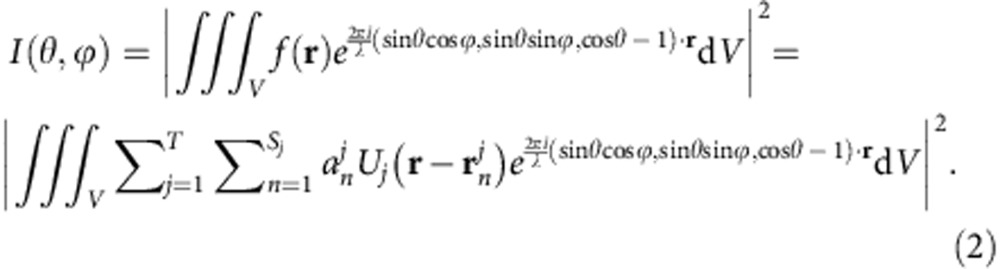


Here, *I*(*θ*, *ϕ*) is proportional to the intensity of the electro-magmatic waves as a function of the angles in spherical coordinates, measured relative to the incident wave direction (*z*), and **r** is the coordinate vector. The proportionality coefficient and further details are provided in the Supplementary Information section (equation (1) there). The integration is taken over the volume defined by the spatial extent of the object (*V*).

### Problem formulation

To set up the problem as a sparse recovery problem, we define a 3D grid (of *M* sites) for the possible positions of each atom, repeating the grid for *T* different elements separately. We arrange the unknowns in a vector 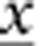 (where, 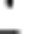 represents a ‘numeric column vector': a series of values), whose entries are 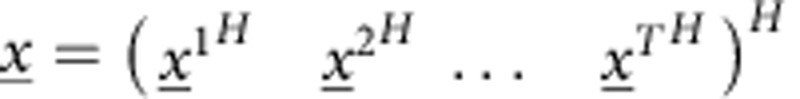 (where the superscript *H* represents conjugate transpose). Here 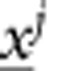 is the vector of unknowns, of size *M*·*T*, associated with element *j* described on the *M* grid sites. The *m*-th entry of 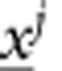 is 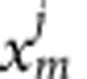, where 
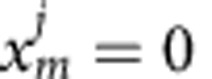
 if no atom of element *j* resides at site *m*, while 
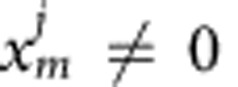
 means that such an atom resides at this site. The measurement vector is denoted by 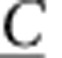, where the value of the *l*-th entry, *C*_*l*_, is proportional to the intensity at angles *θ*_*l*_ and *ϕ*_*l*_ (*C*_*l*_=*I*(*θ*_*l*_, *ϕ*_*l*_)), with *L* being the total number of measurements, namely, of the intensity readings in the detectors. The measurement of the *l*-th detector is 
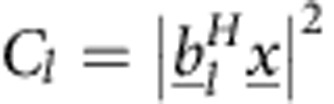
, where the vector 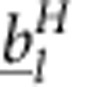 represents one (vector) term in the transfer function of the system, 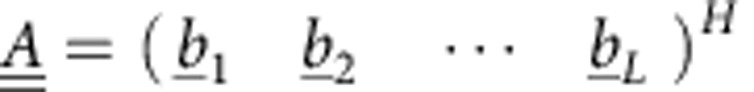, which is simply the 3D Fourier transform operator measured on the Ewald sphere.

With this notation, our mathematical problem can be described as follows:


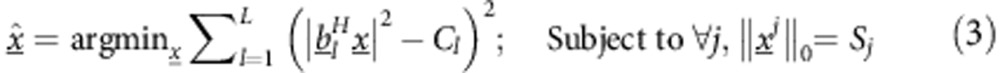


where
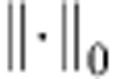
 is the *L*_0_ norm which counts the number of non-zero entries in the vector. Here, *S*_*j*_ is the number of atoms of the *j*-th element, which is assumed to be known from stoichiometry. Note that equation (3) is a difficult problem to solve—there is no guarantee for a unique solution, and furthermore, no assured method to find a global minimum. This is where the power of the sparsity assumption comes in: the fact that the solution is known to be sparse, (that is, that *S*_*j*_ are small) allows us to utilize recently developed methods that solve sparse quadratic problems such as this one. Specifically, to find a sparse solution to equation (3), we use the GESPAR^35^ method with the set of matrices 
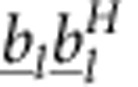
 relevant to our problem. Sparsity-based Ankylography requires some modification to the formulation in ref. 35. Our algorithm is described in detail in the Methods section.

### Comparing our sparsity-based technique with the HIO algorithm

**Figure 2:**
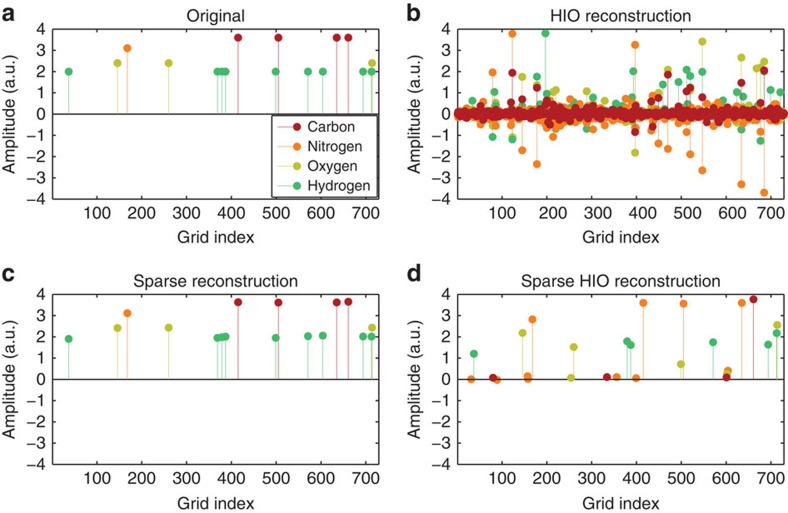
Ankylographic reconstruction of the threonine molecule. (**a**) True 3D positions of the atoms of threonine, displayed in a one-dimensional grid index. Each atom is marked by a circle coloured according to its type (carbon, nitrogen, oxygen or hydrogen), with the vertical axis marking the amplitude, which reflects the charge density in that type. (**b**) The reconstructed molecule using the HIO algorithm taken from ref. 10. (**c**) Our sparsity-based reconstruction using the GESPAR algorithm. (**d**) Reconstruction with HIO while enforcing sparsity in the algorithm.

**Figure 3:**
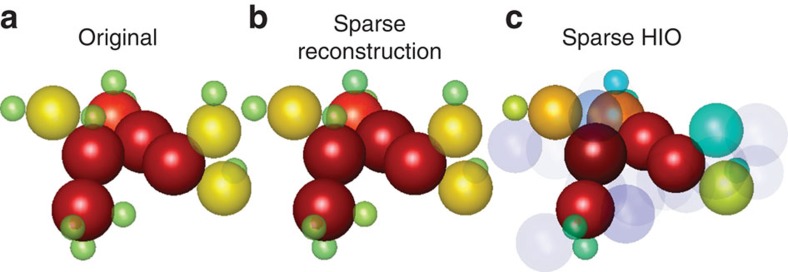
Ankylographic reconstruction of the 3D structure of the threonine molecule. (**a**) The original 3D structure of threonine. (**b**) Sparse reconstruction using our sparsity-based method, GESPAR. (**c**) Sparse reconstruction performed by introducing sparsity into the HIO algorithm used in ref. 10. Adding the sparsity constraints to HIO leads to major improvement in the reconstruction, but the recovery quality is still inferior compared with GESPAR. Using sparsity-based methodology facilitates correct reconstruction, as shown in (**b**). The different radii of the spheres represent the atoms, from largest to smallest, carbon, nitrogen, oxygen and hydrogen. The colours represent the charge density of the atoms, from highest to lowest (red, orange, yellow, green, blue and light purple). Specifically, the light purple spheres in **c** are artifacts of the HIO algorithm.

A typical example is shown in Figs 2a and 3a, where we simulate the recovery of the 3D structure of the amino acid threonine. This molecule, sketched in Fig. 1 and displayed more clearly in Fig. 3a, has 17 atoms: four carbons (red spheres), one nitrogen (orange sphere), three oxygens (light green spheres) and nine hydrogens (dark blue). For clarity, we plot the 3D structure streamlined sequentially, and assign to it a one-dimensional grid index as shown in Fig. 2a. In this example, the position of each of the atoms is marked by a circle of its associated colour on the 9^3^ grid, where the grid is considerably denser than the radius of the smallest atom (see Supplementary Information). The vertical axis provides the amplitude, which reflects the effective charge density associated with each atom.

First, we test the ability of the current Ankylography algorithm (used in Ref. 10) to recover the 3D structure of threonine. To do that, we use a slightly modified version of the algorithm used in ref. 10 and available at http://www.physics.ucla.edu/research/imaging/Ankylography/index.htm. Essentially, this is the standard hybrid input–output (HIO) method^22^,^23^, which is commonly used for phase retrieval. As a model for the sought information, we insert the set of hovering spheres (defining threonine) into the algorithm. The HIO algorithm is basically iterating Fourier transforms back and forth between the object and the Fourier domains, using the measured data (absolute value of the 3D Fourier transform), and applying prior knowledge on the ‘support' of the object (the known region within which the molecule resides). When we attempt to use the HIO algorithm for Ankylography as in ref. 10, we have to represent the 3D information with 55^3^ voxels (volume pixels), for the sake of sufficient resolution. This attempt to reconstruct the 3D structure of threonine has completely failed: as argued in the Comment^26^ and Reply^27^, this method is not expected to work because the number of voxels greatly exceeds 32X32X20, which is the current state of the art in Ankylography. Following this unsuccessful attempt, it is natural to try using the HIO algorithm with the additional prior information that the object (the molecule) can be represented as a set of spheres. The result is shown in Fig. 2b: this attempt also fails, in spite of the additional prior information. The HIO algorithm converges to a solution occupying all the possible number of d.f. (that is, the grid in Fig. 2b is fully populated), which is clearly an erroneous solution.

This is where sparsity makes the big difference. In sharp contrast to the other attempts, our sparsity-based GESPAR algorithm provides excellent reconstruction, as shown by the reconstruction on the streamline grid in Fig. 2c, and by the visual 3D plot of Fig. 3b. See further details on the algorithm in the Methods section. Clearly, sparsity-based Ankylography can recover the 3D structure of molecules of much greater complexity and details than ever anticipated from Ankylography.

It is essential at this point to elucidate the general role of sparsity (rather than the specific algorithm), in our successful reconstruction, where the standard HIO algorithm fails. To do that, we add sparsity to the HIO algorithm, as prior information. More specifically, in every iteration of the HIO algorithm, we enforce the 3D image to be sparse under the underlying basis functions (the set of spheres) by thresholding the coefficients^48^. The result is shown in Figs 2d and 3c. Examining the result, it is clear that adding sparsity constraints to the HIO algorithm results in a huge improvement, but the reconstruction is still poor. Clearly, GESPAR outperforms the sparse HIO approach, consistent with ref. 35.

Following this example, and many other examples we have simulated, several conclusions can be drawn. First, Ankylography features a small number of d.f., hence it is amenable to algorithmic methods relying on sparsity. Second, our current sparsity-based phase-retrieval algorithmic methodology enables the recovery of the 3D structure of molecules occupying two orders of magnitude more voxels than what Ankylography can handle without sparsity^10^. In fact, our sparsity-based method has no upper limit on the size of the molecules. Last but not least, we emphasize that it is indeed the sparsity concept making this recovery possible, as we have shown that adding sparsity to standard methods considerably improves their performance. Altogether, it is clear that sparsity significantly improves Ankylography, making it a highly promising method in the next generation of structural biology experiments.

### Performance of our sparsity-based Ankylography algorithm

**Figure 4:**
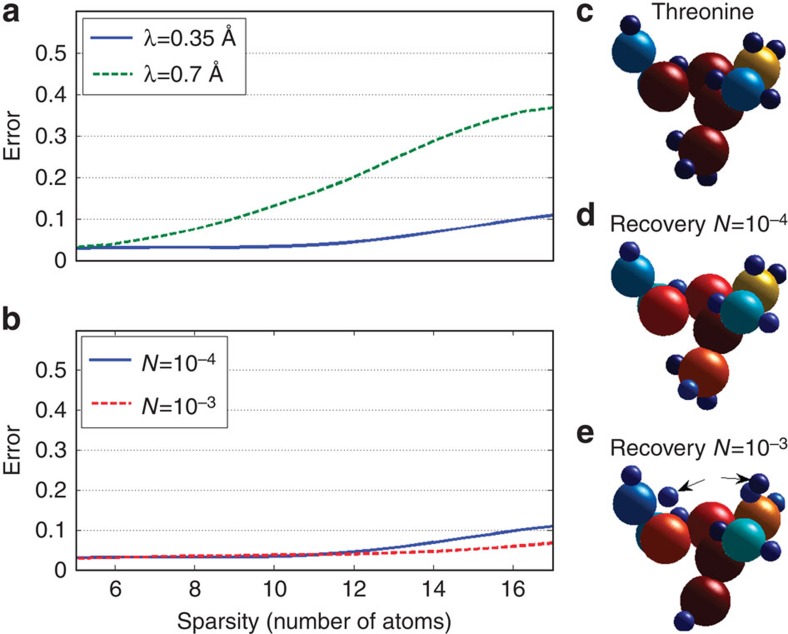
Performance of our sparsity-based algorithm. The plots show the reconstruction error (defined in equation (4)) as a function of sparsity (number of atoms in the molecule), (**a**) for different wavelengths, blue for wavelength 0.35Å and dashed green for 0.7Å (with a given noise level of N=10^−3^), and (**b**) for various noise levels at an optical wavelength of 0.35Å. The resolution of the 3D structure (smallest distance between the centre positions of two spheres) is 1 Å. The plots on the right demonstrate two examples of reconstruction of the threonine molecule (**c**), at 0.35 Å wavelength and under two different noise levels: (**d**) *N*=10^−4^ and (**e**) *N*=10^−3^. Notice that, in (**e**), two of the small blue spheres are misplaced. At *N*=10^−4^ the reconstructed 3D structure is practically indistinguishable from the true structure of threonine. The atoms are represented by the spheres of different radii and the colours represent the charge densities, as in Fig. 3.

With noise robustness being a major concern regarding the performance limits of Ankylography, it is essential to study the performance of our technique in a statistical fashion, in terms of the level of sparsity and permissible noise levels. To do that, we test our algorithm on 600 examples, under different conditions of signal-to-noise ratio (SNR) and optical wavelength. Importantly, we examine the algorithm in a realistic scenario, where the molecule is not restricted to any particular grid, while the recovery is made on a fine 3D grid (four 121^3^ basis functions), such that the radius of the smallest atom (hydrogen) is three times larger than the grid unit. Further details on these simulations are provided in the Methods section. The results are shown in Fig. 4, which displays the reconstruction error as a function of sparsity (total number of atoms). Here, the normalized reconstruction error (a number between 0 and 1) is defined as


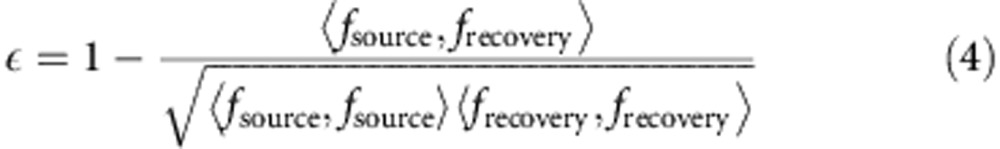


where *ε* is the error, *f*_source_ is the original 3D image (defined above), *f*_recovery_ is the image recovered from the 2D intensity pattern given by equation (2), and 
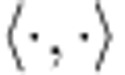
 is the inner product operator. In these simulations, we use white noise (added to *I*(*θ*,*ϕ*)) distributed uniformly on the sphere defining the measured data (assuming the noise originates from isotropic volume scattering). The noise level, *N*, is defined as the fraction of noise to the total power of the scattered light (the measurements). The values we use in the simulations yield SNR that is much smaller than the SNR taken in ref. 10. Figure 4a shows the reconstruction error as a function of sparsity for three wavelengths. Expectedly, the performance is better at shorter wavelengths, which yields higher resolution. Figure 4b shows the reconstruction error as a function of sparsity for various noise levels at wavelength of 0.35 Å. This wavelength is chosen such that it corresponds to 1/3 of the finest resolution of our information (the smallest distance between centres of spheres). The conclusion drawn from these figures is that our method work well under realistic conditions. For example, for a noise level of *N*=0.001 and *λ*=0.35 Å, the algorithm performs well as long as the total number of atoms is smaller than ∼20.

## Discussion

The simulations indicate that increasing the SNR is of major importance. The challenge in doing that is the photon flux at short X-ray wavelengths. The current XFEL emits ∼10^12^ photons at every pulse; however, numerical simulations have indicated that the combination of self-seeding and undulator tapering techniques can increase the pulse intensity by two orders of magnitude^49^. Furthermore, since photons are bosons, there is no fundamental limit on the pulse intensity, and it is expected that the intensity of XFEL will continue to increase as new techniques are being developed. As such, the SNR within which our sparsity-based method can recover structures of single molecules is within reach in the near future. Importantly, proteins have large scattering cross-sections, scattering more photons than a single amino acid, which makes our method viable especially for proteins (which contain multiple amino acids) with the present technology.

**Figure 5:**
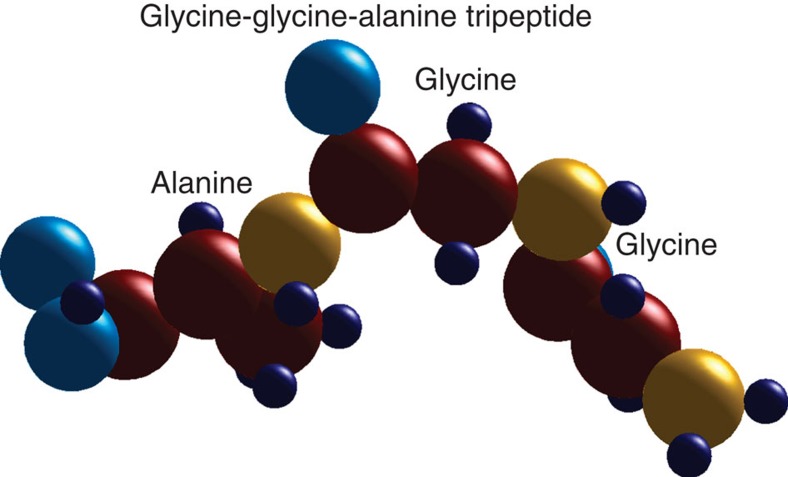
Sparsity-based reconstruction of a glycine–glycine–alanine tripeptide. The plot shows a glycine–glycine–alanine tripeptide, which consist of three amino acids two glycine and one alanine. The simulated reconstruction procedure employs 1 Å wavelength, by using a basis of amino acids, which has freedom of translation and freedom of rotation. The original 3D structure of this molecule looks virtually identical to the reconstruction, to within minute errors in the reconstructed positions (up to a few %). The atoms are represented by the spheres of different radii and the colours represent the charge densities, as in Fig. 3.

Finally, we note that our sparsity-based method is demonstrated here for the case where the signal sparsity corresponds to a small number of atoms. However, the method is applicable for much more general scenarios—for example, when the sparsity is in the number of amino acids, as is the case for many proteins of interest. In this case, the different ‘building blocks' to be localized and oriented are the known amino acids from which the protein is composed, optimally—along with their possible conformations. Figure 5 shows the reconstruction of the 3D structure of a peptide molecule which is a combination of amino acids with peptide bonds. The molecule is a tripeptide and is composed by two glycine and one alanine amino acids. To reconstruct this structure, we use our sparsity-based procedure implemented on the basis of amino acids that spans all positions and rotations. Importantly, using additional prior knowledge on the amino acid bonds (protein conformation such as Ramachandran plot^50^) and assigning a binary value for every basis element (instead of determining their amplitude) further reduces the number of d.f. dramatically, and can allow the reconstruction of significantly larger structure than what we show in Fig. 5. Our method is actually expected to perform much better for large proteins because these have larger cross-sections, and therefore scatter more photons and increase the SNR. Moreover, the recovery of structures made of large basis elements is possible with a longer wavelength, hence, amino acids of typical size of several angstrom will require the wavelength to be of the same order, up to 10 times larger than for Figs 2, 3, 4, where the X-ray laser technology is more mature.

In conclusion, we suggest a new approach to recover the 3D structure of molecules using Ankylography. Our sparsity-based methodology enables deciphering 3D structures of bio-molecules in a single-shot X-ray laser pulse, and exceeds the current limit of recovered information by orders of magnitude. We have demonstrated the reconstruction of a single amino acid and of a tripeptide, with the recovery methodology implemented on the basis of amino acids. These examples highlight the strength of the sparsity-based Ankylography concept and also demonstrate that it is actually easier to apply it to larger objects. The last example proves the generality of sparsity-based Ankylography and provides an avenue for the future of structural biology. With that, sparsity-based Ankylography can reach the level it can overcome the current bottleneck of structural biology.

## Methods

### Mathematical formulation

Our mathematical problem amounts to construction of a 3D sparse signal from the Fourier magnitude on the Ewald's sphere.

Of course, when the majority of the information is lost, precise reconstruction is not possible, unless we have, or may assume, some additional information about the sought signal. In fact, the problem is even more difficult as the measurements contain noise. We assume that the scattered electro-magmatic field can be approximated adequately (hereafter, this relation is denoted by ≅) by means of known generating functions describing spheres *U*_*j*_(**r**) of radius *R*^*j*^. In other words, we want to reconstruct a 3D optical image assuming that it is comprised of a small known number of (different) spheres of known radii, as described in the Results section. Every kind of sphere *U*_*j*_(**r**) (identified by its radius *R*^*j*^) corresponds to a different atomic element (*j*, in this case), where the elements are known, and also how many atoms are there of each element. We emphasize that the reconstruction is done on a 3D grid, while the spheres themselves do not need to reside on any grid at all: they reflect the actual structure of the molecule which does not necessarily reside on a known grid.

As we already mentioned, the input to the algorithm is the 3D Fourier magnitude, sampled on the Ewald sphere. The output of the algorithm should be the positions of all of the atoms, and their corresponding radii.

Mathematically, the molecule is defined in equation (1), which in the spatial frequency domain yields


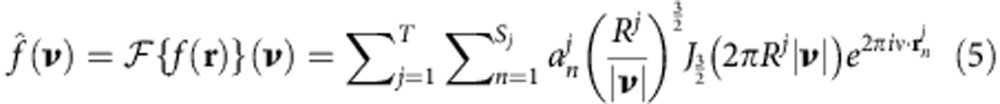


where, 
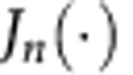
 is Bessel function of order *n*. We define 
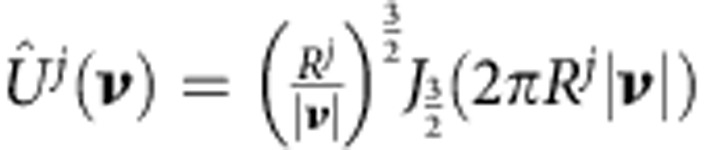
 as generating functions in the frequency domain.

As described in the Results section, we define a 3D grid (of *M* sites) by the set 
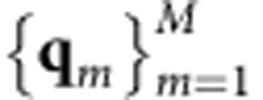
 for the possible positions of each atom for *T* different elements, and a set of sampling points (spatial frequencies) 
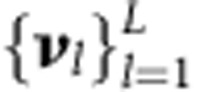
 (related to the angles on the Ewald sphere), defined as 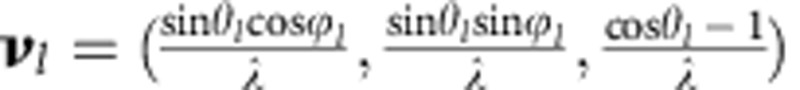. We arrange the unknowns in a vector 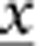 (of size *M*·*T*), whose entries are 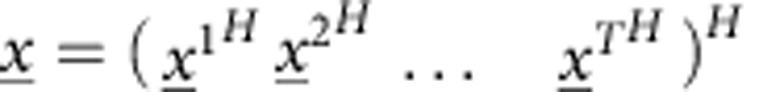 (where the superscript *H* represents conjugate transpose), where 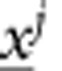 is the vector of unknowns associated with element *j* described on the *M* grid sites. Here, the *m*-th entry in 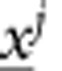 represents the amplitude of *j*-th element at the *m*-th site. Note that 
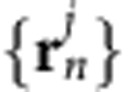
 not necessarily reside on the grid 
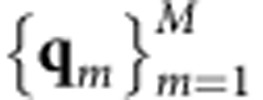
. But if it is on the grid, then we can represent 
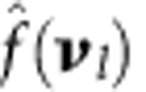
 as an inner product. To do that we define the vector 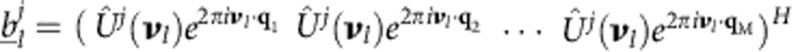 and 
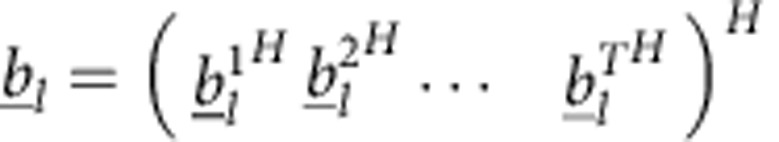
. The measured signal is therefore


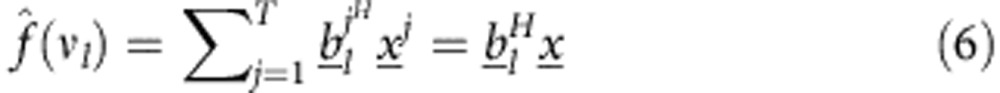


While the sensing matrix is


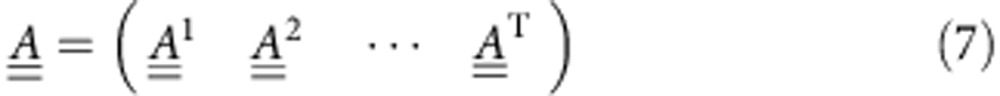


where, 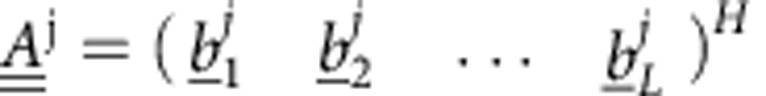. The rows of 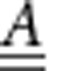 relate to sampling frequencies (total of *L* rows) and the columns correspond to different elements (total *M*·*T* rows). For example, the (*l*, *M*+11) entry, 
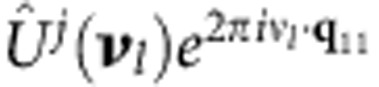
, is related to a sphere of element #2 located at **q**_11_ and sampled at the spatial frequency ***ν***_*l*_. The measurements vector is denoted by 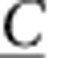, where the value of the *l*-th entry, 
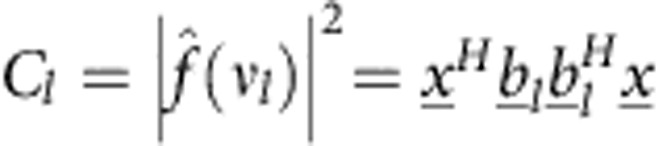
.

Now that we have mathematical representation of the measurements, we consider additional (spatially independent) white noise.


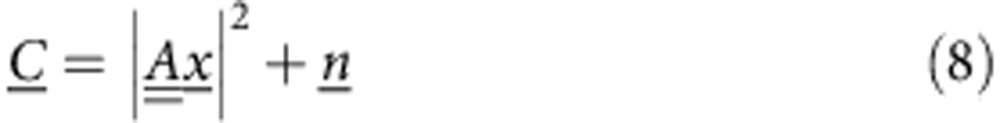


The noise level, 
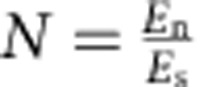
, is the fraction of the noise power to the total power of the scattered light in the measurements surface, where 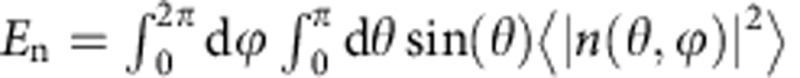 (where 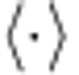 is the expectation value) and 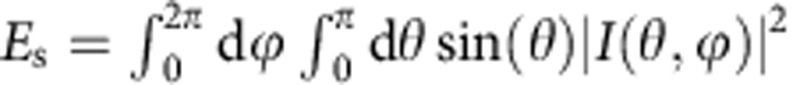. The signal power taken here also includes the scattered field at small angles *θ* (low spatial frequencies on the Ewald sphere), which cannot be measured (because the detectors at those angles are saturated by the incident light beam) but carry most of the energy. The values we use for the noise in the simulations yield SNR that is much smaller than the SNR taken in^10^, yet, as shown in the Results section, our sparsity-based approach is able to recover the 3D structures much better and with information capacity larger by orders of magnitude.

Technically, we seek the vector 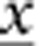 that conforms to the measurements (equation (9)), and at the same time has a known number of units of each element, for example, five atoms of the element carbon. We define the objective as


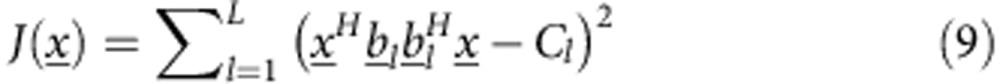


and solve the following optimization problem:


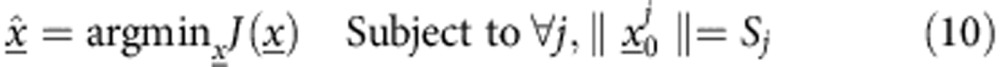


For the sake of further use, the derivative of the objective is calculated below.


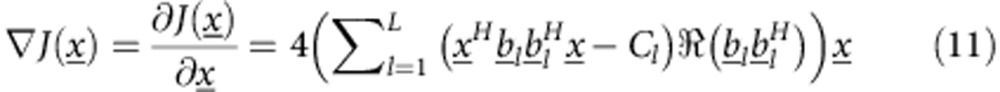


### Description of the algorithm

In order to solve this problem, we use a modified version of a new efficient (greedy) technique for sparsity-based phase retrieval, called GESPAR. The recovery of the unknown vector from the set of equations in equation (9) is an ill-posed problem. However, we have the prior information that our input signal is sparse. Relying on recent work^35^,^39^,^41^,^42^ dealing with the similar problem of finding sparse solutions to the phase-retrieval problem (which constitutes a quadratic compressed sensing problem)—we employ the GESPAR algorithm presented in^35^. GESPAR was originally intended to solve the sparse phase-retrieval problem of recovering a sparse signal from measurements of its Fourier magnitude, but it can also be used to solve the more general sparse quadratic problem^35^.

In order to find a sparse solution to equation (1), we use GESPAR with the set of matrices 
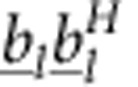
. The algorithm requires modification to the formulation in^35^ (in addition to defining 
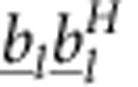
 to correspond to our system). The stages in sparsity-based Ankylography are summarized below (for a more detailed description of the GESPAR algorithm see^35^):

Algorithm: Ankylography GESPAR

Input: Measurements 
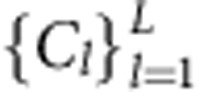
 and sampling matrices 
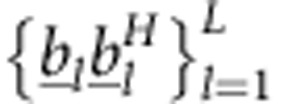
.

Initialize: Set empty support 
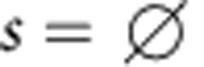
 and initial guess 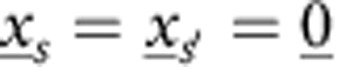.

Loop: while, the cost function is improved (that is, 
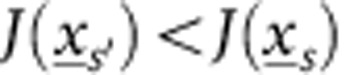
) or support requirement is not satisfied yet (that is, 
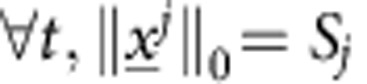
) do

Support update:

Given the support *s*, minimizing 
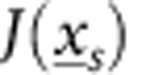
 reduces to a nonlinear least-squares problem, which we solve by the damped-Gauss–Newton algorithm^51^ commonly used for this type of problems. The damped-Gauss–Newton procedure produces an estimate 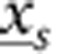.

Perform a local search, an index *k*_*j*_ of element *j* containing a high absolute gradient value. Add *k*_*j*_ to the support 
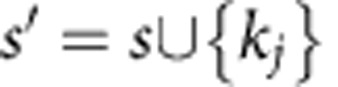
 and perform a damped-Gauss–Newton procedure, given the new support and calculate the cost function 
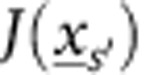
.

Add one atom of the element that minimizes the objective 
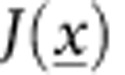
 the most, and that at the same time satisfies 
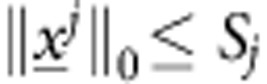
.

Index swapping:

Calculate the cost function gradient 
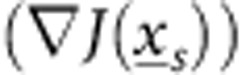
 around the current estimate.

Perform a local search by index swapping, an index *i*_*j*_ of element *j* from the support containing a small absolute valued element with an index *k*_*j*_ of element *j* containing a high absolute gradient value, where the gradient is calculated after zeroing the index *i*_*j*_. This step differs from GESPAR because of the correlativity of the different entries in sparsity-based Ankylography where originally the bases functions in GESPAR are orthogonal. Perform a damped-Gauss–Newton procedure for the support 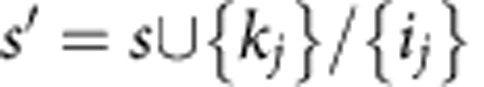 and calculate the cost function 
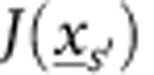
.

Go over all the different elements, *j*=1, 2, 3…*T* and find the support 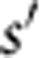 that minimize the objective 
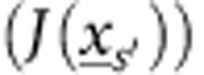
 the most and substitute it as the new support *s* and 
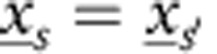
. If the Index swapping step succeeded do it again.

Output: The estimated locations and amplitudes 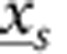.

The difference between GESPAR and our sparsity-based Ankylography algorithm is that our problem contains constraints on the sub-vector 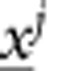, which GESPAR does not have. Consequently, we apply GESPAR to every sub-vector 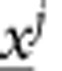 separately and select the best choice. Another difference is that we calculate the gradient of the cost function after zeroing for every index *i*_*j*_.

## Additional information

**How to cite this article:** Mutzafi, M. *et al.* Sparsity-based Ankylography for Recovering 3D molecular structures from single-shot 2D scattered light intensity. *Nat. Commun.* 6:7950 doi: 10.1038/ncomms8950 (2015).

## Supplementary Material

Supplementary Information Supplementary Methods and Supplementary References
