# Supplementary material for: Sparsity-based Ankylography for Recovering 3D molecular structures from single-shot 2D scattered light intensity
Source: Nat Commun. 2015 Aug 20;6:7950. doi: 10.1038/ncomms8950 (PMC4560757; doi:10.1038/ncomms8950)
Supplement: Supplementary Information — Supplementary Methods and Supplementary References [file ncomms8950-s1.pdf]

Supplementary Information

Supplementary Methods

Physical setup

In the simplest form, our system consists of a coherent plane wave incident upon a 3D object (e.g., a molecule). The intensity of the light (proportional to the photon flux) scattered from the object is sampled on a sphere centered on the specimen, as demonstrated in Fig. 1. In reality, in Ankylographic experiments the incident light is in the form of an ultrashort pulse, but the ratio between the bandwidth and the mean frequency is often very small. For example, for X-ray Free-Electron-Lasers (XFEL) this ratio is $\frac{\Delta\lambda}{\lambda} \approx 10^{-4}$, hence the light can be treated as monochromatic at its mean frequency. The electric field amplitude of the wave scattered from the 3D object is calculated through the Born-Oppenheimer approximation[1] , and the field distribution on the surface of the sphere in the far-field of the object is found according to the Huygens-Fresnel principle[1].

To do that, we consider an incident EM (Electro-Magnetic) continuous wave of complex amplitude $E_0 e^{i\frac{2\pi}{\lambda}z}$ (where λ is the wavelength and z is the propagation direction of the incident wave), launched onto a 3D object whose structure is described by the scalar function $f(\mathbf{r})$. The 3D object is of typical size D (for complex molecules D~100nm), while the measurements are taken on a sphere of radius R measured from the center of the object (see Eq. 2). Typically, R is large enough (tens of mm^2) such that the field on the measurements sphere corresponds to the optical far-field (3D Fourier transform, see Eq. 1) of the 3D optical field scattered from the object contained in a volume of ~D^3. Therefore, the scattered field is (same as Eq. 2 in the paper, but with the appropriate constants and scaling of coordinates):

$$E(\mathbf{R}) = \frac{iE_0}{D\lambda}\iiint_V f(\mathbf{r}) \overbrace{e^{\frac{2\pi i}{\lambda}z}}^{Born\ app.} \overbrace{\frac{e^{\frac{2\pi i}{\lambda}|\mathbf{R}-\mathbf{r}|}}{|\mathbf{R}-\mathbf{r}|}}^{Huygens} dV \cong \frac{iE_0 e^{\frac{2\pi i}{\lambda}R}}{D\lambda R}\iiint_V f(\mathbf{r}) e^{\frac{2\pi i}{\lambda}(\hat{z}-\hat{R})\cdot\mathbf{r}} dV = \frac{iE_0 e^{\frac{2\pi i}{\lambda}R}}{D\lambda R}\hat{f}\left(\boldsymbol{\nu} = \frac{\hat{z}-\hat{R}}{\lambda}\right) \quad (1)$$

where, $\mathbf{r}$ is the coordinate, V is the volume, $\mathbf{R} = R\hat{R}$ is the detector direction , $\hat{f}(\mathbf{v})$ is the 3D Fourier transform of $f(\mathbf{r})$, and $\mathbf{v} = \frac{\hat{z}-\hat{R}}{\lambda}$ defines the spatial frequencies on the Ewald sphere, where $\hat{z}$ is a unit vector in the propagation direction.

The approximation used in Eq. 1 is

$$\frac{2\pi}{\lambda}|\mathbf{R}-\mathbf{r}| = \frac{2\pi}{\lambda}\sqrt{|\mathbf{R}-\mathbf{r}|^2} =$$

$$\frac{2\pi}{\lambda}R\sqrt{1-2\frac{\mathbf{R}\cdot\mathbf{r}}{R^2}+\frac{|\mathbf{r}|^2}{R^2}} \overset{R\gg D}{\cong} \frac{2\pi}{\lambda}\mathrm{R} - \frac{2\pi}{\lambda}\hat{R}\cdot\mathbf{r} + O\left(\frac{2\pi|\mathbf{r}|^2}{\lambda R}\right) \quad (2)$$

Where $R \gg D,\ R \gg \frac{D^2}{\lambda}$ implies that $\frac{2\pi|\mathbf{r}|^2}{\lambda \mathrm{R}} \ll 2\pi$, thereby enabling the approximation used in Eq. 1.

The scattered field $E(\mathbf{R})$ has amplitude and phase, where clearly the phase carries most of the information about the 3D object[3]. In principle, it is possible to measure the actual field, amplitude and phase, through holography, but this adds complication to any physical system, and it is especially problematic in the x-ray regime. For this reason, we consider measurements carried out by simple detectors (or cameras), which measure only the optical intensity – amplitude of the field squared (Eq. 1). Because the scattered field corresponds to the Fourier transform of the 3D object, the detectors measure the intensity (the amplitude squared) of the Fourier transform on Ewald's sphere[1], which is

$$\mathrm{I}(\theta,\phi) = \left|\hat{f}\left(\frac{sin\theta cos\varphi}{\lambda},\frac{sin\theta sin\varphi}{\lambda},\frac{cos\theta-1}{\lambda}\right)\right|^2 =$$

$$\left|\iiint_V f(\mathbf{r})e^{\frac{2\pi i}{\lambda}(sin\theta cos\varphi, cos\theta cos\varphi, cos\theta-1)\cdot\mathbf{r}}dV\right|^2 . \quad (3)$$

where the Cartesian coordinates, which correspond to the 3D spatial frequencies, $(\nu_x,\nu_y,\nu_z) = \left(\frac{sin\theta cos\varphi}{\lambda},\frac{sin\theta sin\varphi}{\lambda},\frac{cos\theta-1}{\lambda}\right)$ are in the Fourier domain, and the angles θ and φ are the angles in spherical coordinates relative to the incident wave.

HIO with a basis of hovering spheres

Figure 2b describes the attempt to reconstruct the 3D structure of Threonine with the HIO algorithm with the addition of the prior knowledge that the object (molecule) is made of hovering spheres. Consequently, in that comparing we used the spheres as a basis, namely, we represented the object as a linear combination of bases functions

(spheres of 4 types). In what follows we provide the details of the adjustment made to the HIO algorithm [2] for this purpose.

In this simulation (resulting in Fig. 2b) we use a grid of 9^3 sites, and represent the object with 55^3 voxels (volume pixels), for the sake of sufficient resolution. Mathematically, the object is represented as $\underline{I} = \underline{\underline{B}}\underline{x}$. In order to have enough sampling points (on the Ewald's sphere) we have 155^3 voxels in Fourier space (achieved by padding with zeros, as in [2]). The HIO algorithm is basically iterating Fourier transforms back and forth between the object and the Fourier domains. In Fourier space, $\underline{I_F} = \underline{\underline{F}}\underline{\underline{B}}\underline{x}$, at each iteration we use the measured magnitudes at the frequencies where the values are available, while keeping the phase and the magnitudes at the rest of the frequencies (where measurements are not available) fixed. Then, at each iteration, when applying the inverse Fourier transform we enforce the constraints on the vector $\underline{x}$ as $\underline{\hat{x}} = \operatorname{argmin}_{\underline{x}} \left\| \Re\left\{\underline{\underline{F}}^{-1}\underline{I_F}\right\} - \underline{\underline{B}}\underline{x} \right\|$. Essentially, this stage is a least square operation, which takes the simple form $\underline{x} = \left(\underline{\underline{B}}^H\underline{\underline{B}}\right)^{-1}\underline{\underline{B}}^H Re\left\{\underline{\underline{F}}^{-1}\underline{I_F}\right\}$.

Later on in the paper, we compare the reconstruction with GESPAR (Fig. 2c) and with HIO to which the sparsity constraint is added (Fig. 2d). The GESPAR is described in the previous section, while HIO with the sparsity constraint is simply adding thresholding on the vector $\underline{x}$ in every iteration.

It is worth mentioning the differences between our sparsity-based approach and the HIO with the sparsity constraint. The HIO algorithm, is using all the spatial frequencies in each iteration, whereas in our algorithm we use only frequencies on the Ewald's sphere – where measured values are indeed available. Mathematically, we use the matrix $\underline{\underline{A}}$ (the base functions in Fourier space on the Ewald's sphere) instead of the matrix $\underline{\underline{B}}$ which represents the base functions in real space.

Implementation of sparsity-based Ankylography and comparison with other methods

In the simulations, we examine the algorithm where the molecule (the sought information) is not restricted to any particular grid, whereas the recovery, in the

amino-acids reconstruction examples, is made on a fine 3D grid of $4 \cdot 121^3$ basis functions (for the 4 different elements). In physical units, we assume that the sites of the 3D grid are separated by 0.1Å in the range $[-6\text{Å}, 6\text{Å}]$ in all axes, while the covalent radii of the various atoms are $\{R_C = 0.76\text{Å}, R_N = 0.71\text{Å}, R_O = 0.66\text{Å}, R_H = 0.31\text{Å}\}$ for carbon, nitrogen, oxygen and hydrogen, respectively. In every simulation the scattered light is assumed to be distributed over 6400 pixels in the angular spread $\theta \in \left[\frac{\pi}{24}, \frac{\pi}{3}\right]$.

As a last remark in this section, we offer a reasonable comparison between our sparsity-based methodology and the traditional HIO algorithm used in [2], where the information was represented in voxels. In the description of [2], the finest resolution required to describe an organic molecule at atomic resolution (the covalent radius of the hydrogen atom, 0.3Å) is defined by a single voxel. This sets the limits on the maximum size of the molecules that previous methods of Ankylography [2] can handle to 32×32×20 times 0.3Å, which yields objects that are not larger than $\sim 500[\text{Å}^3]$. On the other hand, the example given in Fig. 5 in our article consists of a simulated box of $8000\ [\text{Å}^3]$ with resolution of 0.1 Å. More importantly, however, we emphasize that our sparsity-based method has no upper limit on the size of the molecules (except for practicalities like memory, computation time, etc.). Clearly, compact representation of the sought information is the key for our successful recovery of the 3D structures. Representing the molecules in their natural basis of atoms, or, as we show in the paper, representing peptides in their natural basis of amino-acids, facilitate compact representation and sparsity-based super-resolution.

Supplementary References

1. Born, M. & Wolf, E. *Principles of Optics: Electromagnetic Theory of Propagation, Interference and Diffraction of Light*. (1980).

2. Raines, K. S. *et al.* Three-dimensional structure determination from a single view. *Nature* **463,** 214–217 (2010).

3. Shechtman, Y. *et al.* Phase Retrieval with Application to Optical Imaging: A contemporary overview. *IEEE Signal Process. Mag.* **32,** 87–109 (2014).
